# Data supporting the shedding of larger extracellular vesicles by multidrug resistant tumour cells

**DOI:** 10.1016/j.dib.2016.02.004

**Published:** 2016-02-08

**Authors:** Vanessa Lopes-Rodrigues, Alessio Di Luca, Diana Sousa, Hugo Seca, Paula Meleady, Michael Henry, Raquel T. Lima, Robert O’Connor, M. Helena Vasconcelos

**Affiliations:** a i3S – Instituto de Investigação e Inovação em Saúde, Universidade do Porto, Portugal; b Cancer Drug Resistance Group, IPATIMUP – Institute of Molecular Pathology and Immunology of the University of Porto, 4200-465 Porto, Portugal; c ICBAS-UP – Institute of Biomedical Sciences Abel Salazar, University of Porto, 4099-003 Porto, Portugal; d NICB – National Institute for Cellular Biotechnology, Dublin City University, Dublin 9, Ireland; e Department of Biological Sciences, FFUP – Faculty of Pharmacy, University of Porto, 4050-313 Porto, Portugal; f Department of Pathology and Oncology, FMUP – Faculty of Medicine of the University of Porto, Alameda Prof. Hernâni Monteiro, 4200-319 Porto, Portugal

## Abstract

To date, there are no simple and minimally invasive methods to diagnose MDR. Extracellular vesicles (EVs) are shed by all cells, carry a specific cargo from the donor cells and are present in several body fluids, which means that they can potentially be easily collected from cancer patients and become the source of biomarkers to diagnose cancer. This data article contains a full list of the proteins identified in the EVs shed by an isogenic pair of chronic myeloid leukaemia cells (MDR cells and their drug-sensitive counterparts) by LC/MS/MS analysis, together with their GeneOntology analysis. In addition, it also contains data from protein content analysis and Dynamic light scattering count-rate events of the referred EVs as well as of the EVs shed from an isogenic pair of non-small cell lung cancer cells (MDR cells and their drug-sensitive counterparts). The interpretation of the data presented in this article and further extensive insights can be found in “Multidrug resistant tumour cells shed more microvesicles-like EVs and less exosomes than their drug-sensitive counterpart cells” [1].

**Specifications Table**

**Table:** 

Subject area	Health sciences
More specific subject area	*Cancer Multidrug resistance, Extracellular vesicles*
Type of data	*Tables and figure*
How data was acquired	Optima XE−100 Ultracentrifuge, Beckman Coulter; Nano series Malvern Zetasizer Instrument (Prager Instruments); Ultimate 3000 nanoLC system (Dionex) coupled to a hybrid linear ion trap/Orbitrap mass spectrometer (LTQ Orbitrap XL; Thermo Fisher Scientific).
Data format	*Analyzed*
Experimental factors	*Multidrug resistance*
Experimental features	*Extracellular vesicles (EVs) isolated from multidrug resistant (MDR) cells (overexpressing P-glycoprotein) and from their drug-sensitive counterpart cells were used to obtain the data.*
Data source location	*– i3S – Instituto de Investigação e Inovação em Saúde, Universidade do Porto, Portugal*
*– Cancer Drug Resistance Group, IPATIMUP – Institute of Molecular Pathology and Immunology of the University of Porto, Porto, Portugal*
*– NICB – National Institute for Cellular Biotechnology, Dublin City University, Dublin 9, Ireland*
Data accessibility	*Data within this article*

## Value of the data

• These data describe the quantification of EVs isolated from MDR cells and from their drug sensitive counterpart cells.
• Data regarding the use of LC–MS/MS analysis to compare EVs isolated from MDR and drug-sensitive counterpart cells.
• EVs isolated from MDR and their drug-sensitive counterpart cells could be valuable for future research on the acquisition of MDR phenotype and improving knowledge in the diagnosis of MDR.

## Data

**Fig. 1:**
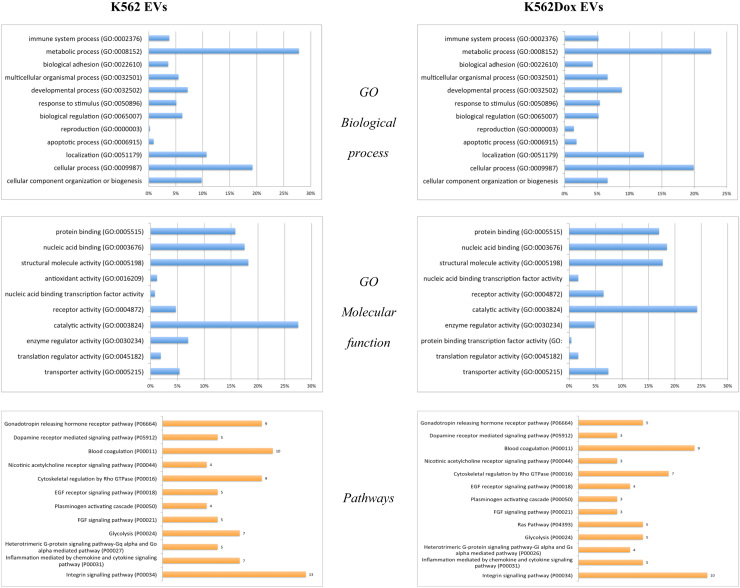
Gene Ontology analysis based on biological process, molecular function and pathway. The analysis was performed in the protein list obtained by LC/MS/MS analysis in the EVs isolated from K562 and K562Dox cells.

The protein content and DLS count-rate events of EVs isolated from MDR and drug-sensitive cells have been shown (Table 1). In Table 2 a list of proteins identified by mass spectrometry, in EVs isolated from CML cells (*K562 – drug-sensitive* cells and *K562Dox – MDR cells*) have been mentioned. Moreover, Fig. 1 shows Gene Ontology analysis performed in the protein list obtained by LC/MS/MS of EVs isolated from K562 and K562Dox cells, based on the biological processes, molecular functions and pathways.

## 1 Experimental design, materials and methods

EVs were isolated from two pairs of isogenic cell lines (MDR and the drug-sensitive counterpart) from two different cancer models, non-small cell lung cancer-NSCLC *(H460 – drug-sensitive cells and RH460 – MDR cells)* [2], [3] and chronic myeloid leukaemia-CML (*K562 – drug-sensitive* cells and *K562Dox – MDR cells*) [4], [5]. Cells were used to isolate EVs, for protein quantification (Table 1), Dynamic light scattering (Table 1) and proteomic experiments (Table 2 and Fig. 1)**.**

### 1.1 Isolation of Extracellular vesicles

EVs were collected from the supernatant of equivalent amounts of cells, cultured in EVs-depleted culture medium. EVs were isolated from these culture media as previously published [6], [1] by various centrifugation steps.

### 1.2 Protein quantification

Protein amount of the isolated EVs was quantified as previous published [1].

### 1.3 Count rate analysis using dynamic light scattering

EVs count rate was measured by dynamic light scattering (DLS), using a Nano series Malvern Zetasizer Instrument (Prager Instruments) as previously published [1].

### 1.4 Sample preparation and mass spectrometry

Pellets of EVs were prepared using previously established methods [1]. Nano LC–MS/MS analysis was carried out using an Ultimate 3000 nanoLC system (Dionex) coupled to a hybrid linear ion trap/Orbitrap mass spectrometer (LTQ Orbitrap XL; Thermo Fisher Scientific) [1]. MS data analysis was carried out as previously described [1]. A protein was considered as being identified in the EVs when it was recognized at least in one of the four biological replicate samples.

### 1.5 PHANTER analysis

Proteins identified in the samples of EVs were analysed using GeneOntology (GO) in the PANTHER database to identify biological processes, molecular functions and pathways (http://www.pantherdb.org/).

## Figures and Tables

**Table 1:** Protein content and DLS count-rate events of EVs isolated from MDR and drug-sensitive cells from both models (CML and NSCLC).

	**Protein content (μg)**	**Count Rate (kcps)**
**K562**	9±0.7	167.3±2.28
**K562Dox**	14±1.5	265.9±7.71
**H460**	7±2.2	149.5±9.3
**RH460**	11±2.8	168.4±5.8

EVs were isolated from the two pairs of (MDR and drug-sensitive) cell lines (from CML and NSCLC). Proteins were extracted and quantified. Count rate was determined by DLS. Data refers to protein quantity (μg) and count rates obtained for EVs isolated from the same number of cells. Results refer to µg±S.E. or to kcps±S.E.

## Appendix A Supplementary material

Supplementary material

Supplementary material: Table 2 | List of proteins identified by mass spectrometry in K562 and K562Dox EVs preparations. (This Table is attached as an excel file with the name: Table_2). Table 2 legend. LC/MS/MS analysis allowed identifying in the eight samples, 304 proteins (232 proteins in EVs shed from K562 cells and 207 proteins in EVs shed from K562Dox cells). For each protein, the Uniprot accession number, protein name and molecular weight are indicated. In addition, for each sample, the peptide charge state, scores (mascot and sequest) and coverage % are indicated. Samples 1 to 4 refer to K562 EVs (grey colour) and samples 5 to 8 to K562Dox EVs (white colour). Within each row, the number of peptide, score (Mascot and/or Sequest) and coverage indicate that the respective protein was identified in the sample. N/A (not available) indicates that the protein was not detected. a) Accession number in the UniProt database. b) Molecular weight of the protein. c) Peptide charge state.
